# Manipulation of Magnetic Beads with Thin Film Microelectromagnet Traps

**DOI:** 10.3390/mi10090607

**Published:** 2019-09-13

**Authors:** Vania Silverio, Miguel Amaral, João Gaspar, Susana Cardoso, Paulo P. Freitas

**Affiliations:** 1 INESC Microsystems and Nanotechnologies, INESC-MN, and IN, 1000-029 Lisboa, Portugal; 2 Department of Physics, Instituto Superior Técnico, Universidade de Lisboa, 1040-001 Lisboa, Portugal; 3 International Iberian Nanotechnology Laboratory, INL, 4715-330 Braga, Portugal

**Keywords:** magnetic trapping, thin film planar microelectromagnets, magnetic beads concentration, microfluidic flow, interactive design

## Abstract

Integration of point-of-care assays can be facilitated with the use of actuated magnetic beads (MB) to perform testing in less expensive settings to enable the delivery of cost-effective care. In this paper we present six different designs of planar microelectromagnets traps (MEMT) with four external coils in series and one central coil connected for an opposite direction of manipulation of MB in microfluidic flows. The development of a simulation tool facilitated the rapid and efficient optimization of designs by presenting the influence of system variables on real time concentrations of MB. Real time experiments are in good agreement with the simulations and showed that the design enabled synchronous concentration and dispersion of MB on the same MEMT. The yield of local concentration is seen to be highly dependent on coil design. Additional coil turns between the central and external coils (inter-windings) doubled magnetic concentration and repulsion with no significant electrical resistance increase. The assemblage of a copper microchannel closed loop cooling system to the coils successfully eliminated the thermal drift promoted by joule heating generated by applied current.

## 1. Introduction

The continuous measurement of therapeutic drugs and metabolites is a strong requirement in transplantation surgery, pulmonary and critical care medicine [1]. Drugs of interest are therapeutics with a narrow action window, which regularly pose considerable challenges in initial and ongoing dosing. These drugs are often subject to polymorphic metabolism with considerable inter- and intra-individual variability requiring therapeutic drug monitoring (TDM) [2]. TDM, however, is regularly performed with one blood sample per dosing interval only, with trough-value (i.e., the plasma level of a pharmaceutical product) measured just before the following dosage. On the other hand, because biomarkers are typically present at very low concentrations within complex samples that contain high concentrations of background material, the methodologies need to be highly selective and accurate [3]. Thus, a cluster of biomarker elements will emit a stronger signal than a single element of the same kind, independently of the signal type, leading to an increased reading of the sensor regardless of its accuracy [4].

A microactuator using magnetic beads (MB) conjugated biomarkers as a driver to form clusters or to induce surface binding is a viable approach to concentrate such biomarkers, increasing the sensitivity and the specificity of the test and at the same time avoiding interference before testing [5,6]. The use of MB provides large surface-to-volume ratio. MB can be suitably biofunctionalized and manipulated by magnetic fields [7,8,9,10,11], diminishing the difficulties posed by mixing at low Reynolds numbers or by the high flow resistances at small scales [12]. Furthermore, MB are commercially available in different sizes, magnetic properties and surface coatings, making them very versatile, especially to realize integrated and miniaturized technologies based on small fluid volumes [13,14]. Permanent magnets (PM) are the most common to generate and control a magnetic field, however, if the microactuator working mode is based on a current flowing across planar spiral coils (resistances), the magnetic field can be modulated micromagnetically by varying the current flowing through the device [15]. Moreover, adding permanent magnets to this setup enforces the magnetic fields by around 10 times [16]. 

Electrically actuated microcoils will heat, reducing its efficiency [17,18] and resistive heating can be a drawback if tests need to be performed under strict temperature ranges. As a result, adapted cooling schemes may be necessary to tackle heat dissipation as the current density of the microcoils can reach thousands of A·mm^−2^, and even higher values in short pulses [19].

In this work we present the manipulation of magnetic beads for therapeutic drug monitoring by enclosing magnetic trapping and integrated efficient cooling. The fabrication of the microactuator allow us to direct the magnetic beads between outer coils and a central coil following the design and optimization investigated by numerical analysis. This design empowers a fast and optimized response for the manipulation of MB and consequently is foreseen to be capable of an improved dosage of the respective therapeutical drug.

## 2. Design and Microfabrication 

### 2.1. Trapping Simulation

**Figure 1:**
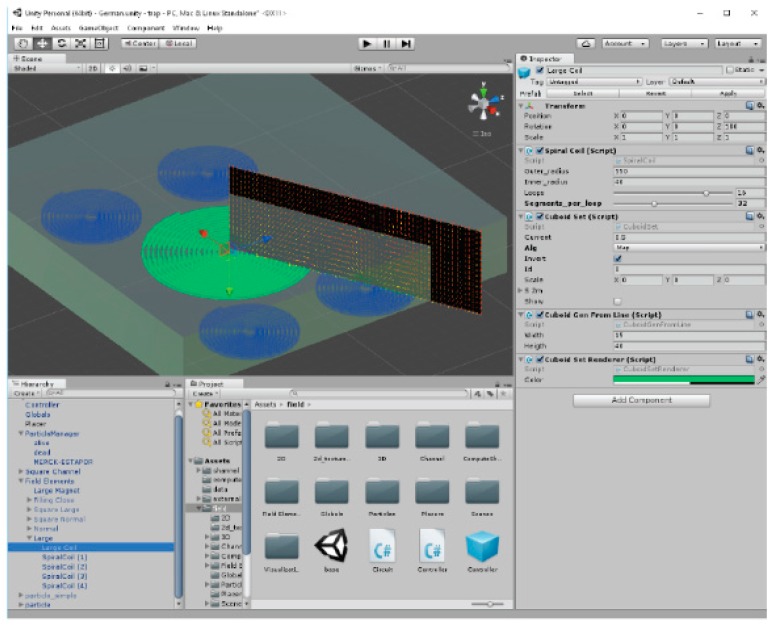
Graphical User Interface of UNITY3D editor (Video S1: multimedia view).

The planar microelectromagnets traps (MEMT) design parameters (thickness, width, number of turns, positioning of coils, etc.) were simulated for enhanced magnetic concentration efficiency. Theoretical prediction of the magnetic and magnetostatic force fields generated by the MEMT by imposing a DC current to the coil tracks was performed with assistance of a homemade software (Figure 1). 

The software developed on the UNITY3D (UNITY version 5.4.2, San Francisco, CA, USA) game development platform [20] was scripted in C# language for an easy-to-use graphical user interface (GUI). The objects were defined and positioned in space, and components such as scripts could be assigned to them. This feature allowed real-time readjustment of the magnetic field solutions deriving from modifications to the design of the MEMT. Each conductor was approximated by a series of cuboids and using a closed-form expression, the field contribution for each cuboid was calculated and added to a total. The magnetic flux density $\vec{B}$ at a point $\vec{r}$ produced by a cuboid, i.e., a parallelepiped of straight angles which passes through a uniform current density $\vec{J}$ was defined as

(1) $$\vec{B}=\;\frac{\mu_{0}}{4\pi }\;\vec{J}\times \;\vartheta P\;\left(\vec{r}\cdot \vec{g}\right)$$

where *µ*_0_ is the magnetic permeability of free space (*µ*_0_ = 4π × 10^−7^ N·A^−2^) and *P* is an operator. The magnetic flux density gradient is then given as

(2) $$\begin{array}{ll} \nabla \vec{B}\; & =\;\left[\partial_{j}B_{i}\right]\;=\;\left[\partial_{j}\vec{B}\right]=\left[\frac{\mu_{0}}{4\pi }\;\vec{J}\times \partial_{j}\vec{U}\;\right]:\\ & =\;\frac{\mu_{0}}{4\pi }\;\vec{J}\times \left[\partial_{j}\vec{U}\right]\;=\;\frac{\mu_{0}}{4\pi }\;\vec{J}\times G\;\left(\vartheta \vec{w},\;\vartheta \vec{l\;}\;\right) \end{array}$$

where $\vec{U}$ is the magnetic potential, and *G* is the matrix (defined by Equation (A13)), linear in width *w* and length *l*. Further details of the definitions, mathematical derivations and pseudo-code for the magnetic flux density and magnetic flux density gradient calculation are given in the Appendix A.

### 2.2. MEMT Microfabrication

**Figure 2:**
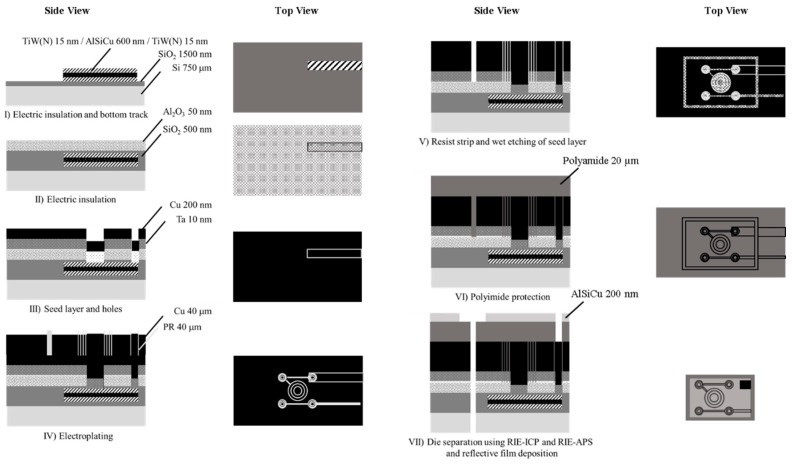
Process flow of the planar microelectromagnet trap (MEMT) fabricated onto the Si wafer (not to scale).

The MEMT chips were microfabricated on top of an 8” single side polished 725 µm-thick Si wafer (Figure 2). An isolation layer of SiO_2_ (1500 nm) was deposited followed by the sputtering of TiW (N) (15 nm)/Al_98.5_Si_1.0_Cu_0.5_ (600 nm)/TiW (N) (15 nm) which defined the bottom track (Figure 2I).

Photoresist ashing was done with an O_2_ plasma. The first lithographic step defined the bottom tracks for subsequent ICP (Inductively Coupled Plasma) using SiCl_4_ and CF_4_. Electric insolation between the bottom and top tracks was achieved with the deposition of SiO_2_ (500 nm). An additional layer of Al_2_O_3_ (50 nm) was sputtered as a stopping layer for a posterior etching of the Ta seed layer (Figure 2II). Holes to connect the bottom and top tracks were defined by lithography, reactive ion etching (RIE) of the Al_2_O_3_ layer (stopping at the SiO_2_ layer) and RIE-APS (advanced plasma system) etching of the SiO_2_ layer (stopping at the TiW (N) layer). Deposition via sputtering of Ta (10 nm)/Cu (200 nm) seed layer (Figure 2III) and patterning of 40 µm-thick photoresist preceded the Cu electroplating (Cu deposit on H_2_SO_4_, 280 min, 5 mA·cm^−2^, 24 °C, 12 rpm agitation) (Figure 2IV). After a resist strip, the seed layer was wet etched (1:2 H_2_O:Al etchant) and the Ta layer removed by XeF_2_ dry vapor-phase etching (Figure 2V). The sample was then spin coated with polyamide (20 µm) for passivation, uniformization of surface topography and structural reinforcement (Figure 2VI). AlSiCu (1 µm)/TiW (N) (30 nm) was sputter coated, lithographed and etched (RIE-ICP) to serve as a hardmask for polyamide etching (RIE-APS) up to the Si substrate. Al_2_O_3_ etching (RIE-ICP), SiO_2_ etching (RIE-APS), and Si etching (DRIE, deep reactive ion etching) followed to individualize the dies (Figure 2VII). 

**Figure 3:**
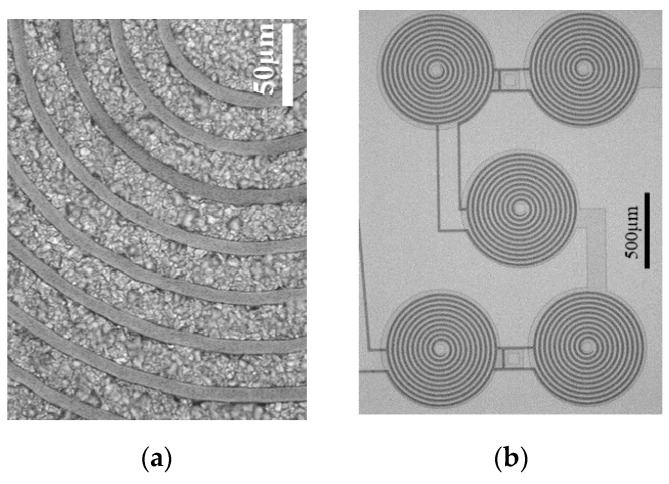
Microscope inspection of the microfabrication process (**a**) detail of the MEMT tracks and (**b**) SCR (S-configuration, CR—Central Reversed) MEMT.

The thickness (40 ± 3 μm), width (20 ± 1 μm) and separation between the tracks (10 ± 1 μm) of electroplated Cu was characterized with a mechanical profilometer before the coating with polyimide (Figure 2VI). Microscope inspection tracked the microfabrication process (Figure 3).

**Figure 4:**
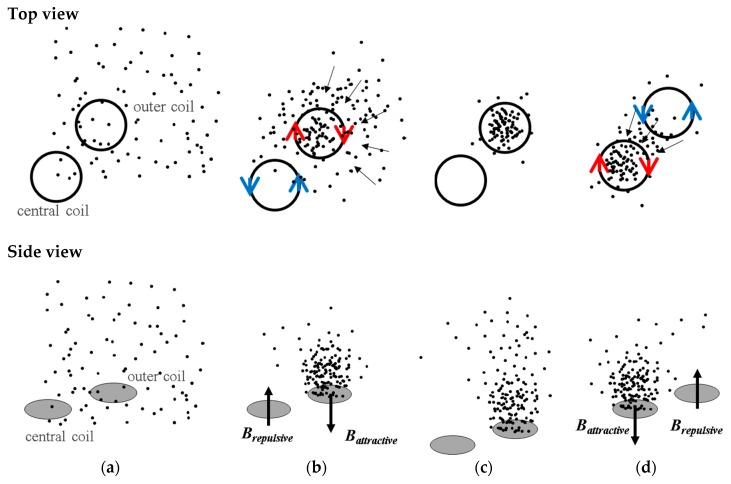
Schematics of ¼ of the MEMT system and magnetic beads (MB) concentration/dispersion process. The red and blue arrows (top views) indicate the current flow while the black arrows (side views) indicate the direction of the magnetic flux density. (**a**) MB are moving freely in the flow with uniform distribution. During the trapping experiments, without flow, the current is set to the coils for the magnetic actuation to start. (**b**) The concentration of MB in the outer coils results from both the inward electromagnetic force generated at the outer coils and the outward electromagnetic force at the central coil. (**c**) Instantaneous interruption of the current. (**d**) When the direction of the current is inverted, and consequently the magnetic forces, the MB previously concentrated over the outer coils are then oriented towards the central coil.

The MEMT designs consist of sets of four external coils in series and one central coil connected for an opposite direction of manipulation (CR—Central Reversed) (Figure 3b). Depending on the current direction one can choose the orientation of the magnetic field, which will impact the force direction. The process is schematized in Figure 4. In different positions of the MEMT, beads are attracted over one coil and repelled over another depending on the direction of the magnetic force.

**Figure 5:**
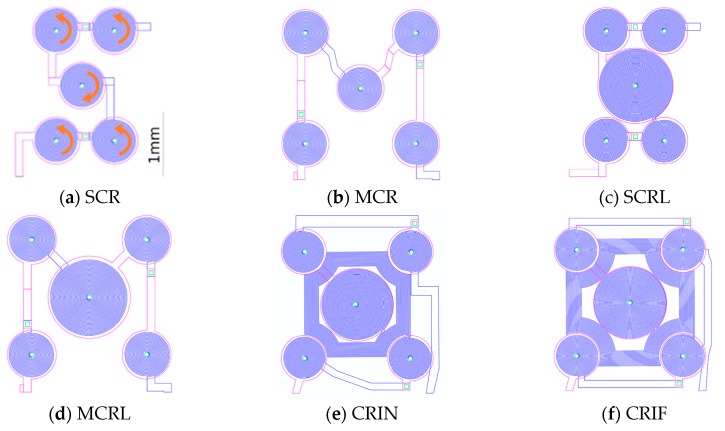

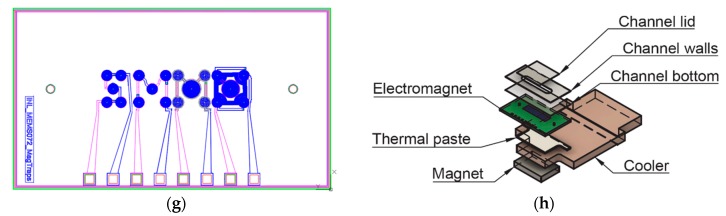
(**a**–**f**) Layout of MEMT configurations, S: S-configuration; M: M-configuration, C: Central, R: Reversed, L: Large central coil, I: Inter-windings, N: Near, F: Far. The current in the central coil is opposite to that in the external coils as indicated by the arrows. The distance between the center of the central coil and the center of the external coils is 0.99 mm for SCR and SCRL whereas for the others is 1.2 mm; (**g**) schematics of one die with 4 different MEMT configurations. From left to right: SCR, MCR, MCRL, CRIF; (**h**) schematics of the MEMT mounting: Permanent magnet, copper microchannel cooler and microfluidic channel for bioflow.

Other sets vary from the first (SCR S-configuration) by changing the placement of external coils from S-configuration to M-configuration (Figure 5a,b); by increasing the size of the central coil (L—large, Figure 5c,d); and by adding inter-windings (I) to the current path between the center coil and the external coils (N—near or F—far, Figure 5e,f). Here, the current flows through the windings in the same direction as in the central coil. The different designs were combined in 8 chips to a total of 56 chips in one 8” wafer. Figure 5g depicts an example of one chip with four different coils. Figure 5h shows the mounting scheme adopted in the experiments. Due to the Joule effect, a copper microchannel closed loop cooling system described elsewhere [8] was implemented together with a pulsed strategy to effectively diminish the MEMT temperature from up to 200 °C to values compatible with biomedical experiments, limited at 38 °C. As an example, activating the SCR MEMT by running a sequence of 500 mA pulses with 8 s duration followed by 15 s interval and operating the cooling system at 100 mL·h^−1^, the surface temperature was maintained between 23.3 °C and 27 °C after 16 min operation [8]. The thermal contact between the chip and the cooler was achieved by means of an intermediate layer of thermal paste (8.9 W·m^−1^·K^−1^, Arctic Silver^®^ 5, Visalia, CA, USA).

The MB used were Estapor^©^ Small Carboxyl-Modified SuperParamagnetic Microspheres, M1-030/40 from Merck Millipore (Burlington, MA, USA), with a stated diameter of 300–500 nm. A diameter of 300 nm was used in all calculations. The magnetic moment per MB was experimentally determined by computer controlled vibrating sample magnetometry (resolution 0.1 Oe, sensitivity 10^−5^ emu, vibrating frequency 200 Hz, DMS 880) to be *m* = 5 × 10^−16^ A·m^2^ and mass 1.83 × 10^−14^ g. The superparamagnetic signature of the Estapor^©^ MB required an external magnetic field to activate their magnetization. This external vertical field was created by a permanent magnet (NdFeB, Q-12-08-02-N, Supermagnete, Hamburg, Germany) positioned below the chip (Figure 5h). The MB were functionalized with fluorescent BODIPY^©^ 515 (Thermo Fisher Scientific, Waltham, MA, USA) probes for visual inspection. The same batch was used in all experiments. 

## 3. Results and Discussion

**Figure 6:**
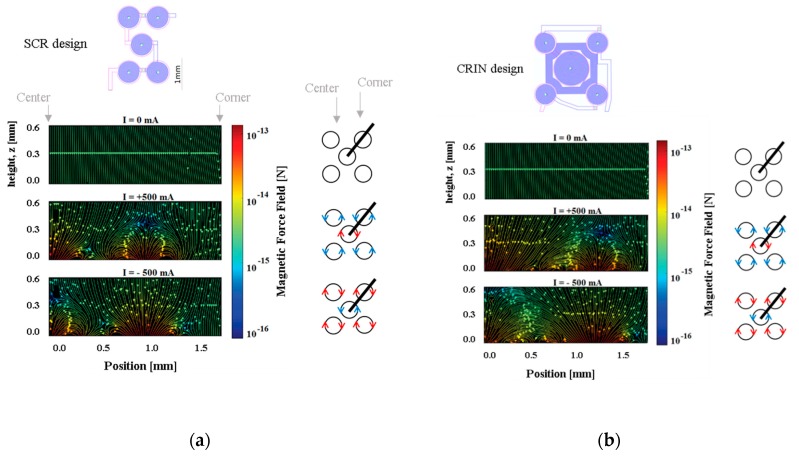
Plots for the (**a**) SRC design. At approximately *z* = 200 µm the force field moves beads between coils, but below *z* = 100 µm the field moves beads between the interior and contour of each coil (**b**) CRIN design. The field becomes effective at moving beads between coils at *z* = 50 µm.

The magnetic force field was simulated for each electromagnet design and plots are presented along the diagonal between the center of the central coil and the center of one of the external coils. Figure 6 presents the resulting magnetic force when actuating the SCR and the CRIN designs with 0 mA, +500 mA, and −500 mA starting at 0 µm from the coil surface up to 600 µm.

Considering the microfluidic channel bottom placed at a distance of 100 µm from the top of the electromagnet, the magnetic beads will move from the edge of the coils to the center, and vice-versa when actuation occurs. However, if the microchannel is placed more than 200 µm above, a region with low forces appears above the lateral coil, which reduces the efficacy of trapping. Beads sufficiently close to the bottom of the channel will be strongly attracted, relative to the rest of the force field, towards one of the sides of the current tracks, following the global pull that can be seen near the top of the plot. The separation between the channel and the electromagnet as well as the current imposed will define the strength of trapping which can be tuned for different types of real operation in immunoassays.

**Figure 7:**
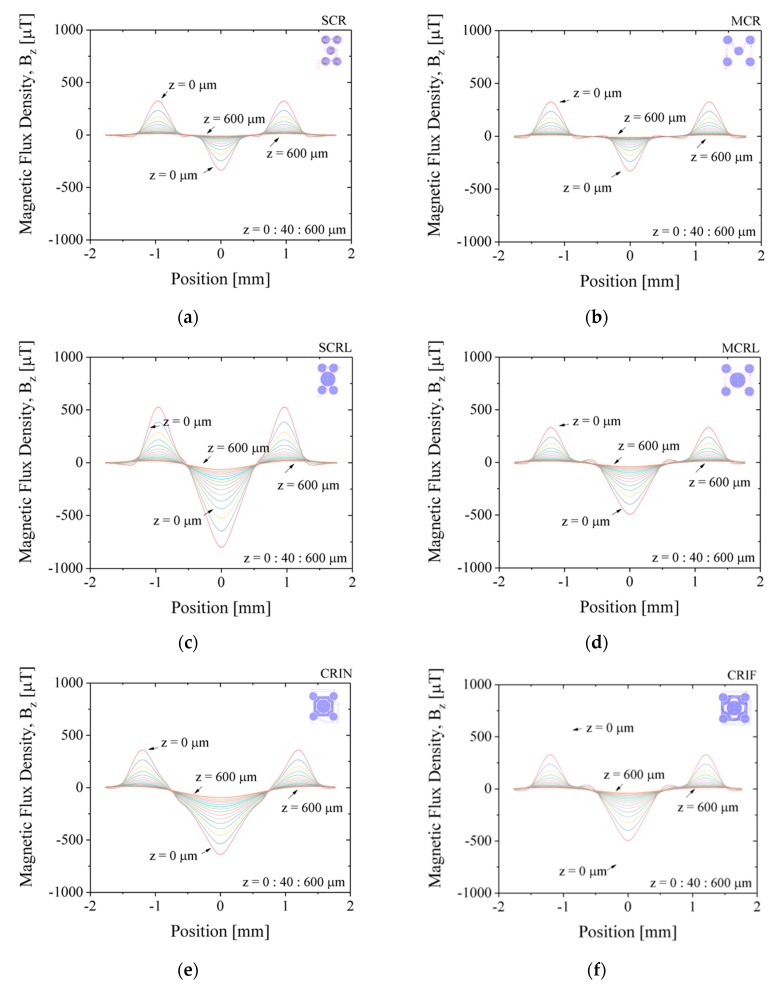
Simulated results for magnetic flux density, *Bz*, along the diagonal of the chip, for *z*-position varying from 0 to 600 µm in 40 µm steps. Applied current of +30 mA. (**a**) SCR, (**b**) MCR, (**c**) SCRL, (**d**) MCRL, (**e**) CRIN, (**f**) CRIF

Figure 7 shows the simulated results for magnetic flux density, *Bz*, along the diagonal of the chip, for z-positions varying from 0 µm (at the surface) to 600 µm away from the surface, in steps of 40 µm. The applied current is set to −30 mA. Positive values of *Bz* will translate into repulsion of MB away from the surface and negative values of *Bz* to attraction of MB towards the surface. The magnetic flux density, *Bz*, is minimum (attraction) at the center of the coil near the surface at z-position equal to 0 µm and decreases intensity further away from the surface up to 600 µm for all configuration, as expected. MEMT with coils of equal size generate *Bz* equal in the central and external coils (e.g., SCR and MCR, Figure 7a,b) unlike larger central coils MEMT, where *Bz* is always stronger in the central coil (e.g., SCRL and MCRL, Figure 7c,d). Larger distances between central and external coils induce secondary variations of *Bz* between the central and the external coils, more pronounced closer to the surface. This is clearly seen in the spacing between coils from −0.8 mm to −0.5 mm and from 0.5 mm to 0.8 mm in Figure 7d. The secondary variations degrade the *Bz* gradient and consequently the yield of MB transition between the central and the external coils. The inclusion of inter-windings was successful in the removal of these secondary variations of *Bz* only for CRIN (Figure 7e), probably enhanced by the lack of free intercoil space. This idea is reinforced by the resulting *Bz* profiles obtained for CRIF (Figure 7f), in which the larger intercoil space seems to be promoting secondary variations of *Bz*. A constant *Bz* gradient between coils is observed for both SCRL and CRIN MEMT (Figure 7c,e), indicating these as preferable candidates for chip integration.

**Figure 8:**
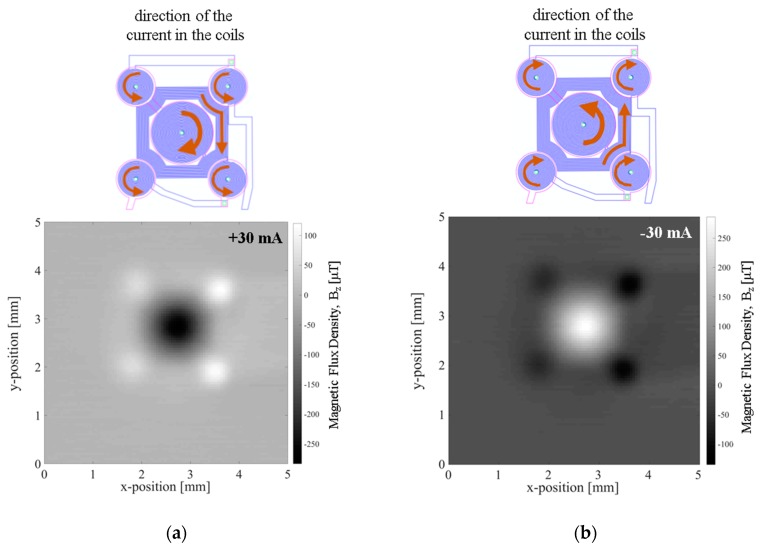
Electromagnetic characterization of the coils using a Magnetic Tunnel Junction (MTJ) sensitivity −26.4 Ω·Oe^−1^, dimension 58.5 × 4 µm^2^. Magnetic flux density generated while imposing a current to the MEMT tracks (**a**) CRIN MEMT, +30 mA, (**b**) CRIN MEMT, −30 mA.

The magnetic flux density in the vertical direction, *Bz*, resulting from imposing 30 mA to the MEMT was measured at 200 µm from the chip surface in a custom made magnetoresistive scanner detailed in [21] making use of a Magnetic Tunnel Junction sensor (sensitivity −0.26 Ω·µT^−1^, dimension 58.5 × 4 µm^2^). The magnetic scan (5 × 5 mm^2^, 0.05 mm step size) shows the vertical attractive magnetic flux density of −283 µT generated in the central coil, opposing that of the external coils (120 µT) when +30 mA currents are imposed to the coil (Figure 8a) and the system reverses when applying −30 mA (Figure 8b).

**Figure 9:**
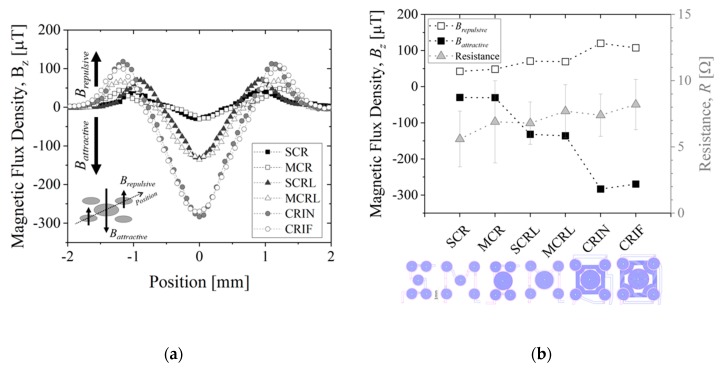
(**a**) MEMT magnetic response along the diagonal. When applying +30 mA current, the maximum of *Bz* is obtained in the external tracks (*B_repulsive_*) while the minimum (*B_attractive_*) is collected in the central coil and (**b**) magnetic flux density and corresponding resistance for each set of coils. Measurements performed at 200 µm from the chip surface.

Figure 9 shows a summary of the electromagnetic characterization of different MEMT designs when applying +30 mA measured at 200 µm from the chip surface and presented for the diagonal of the MEMT. For the configurations studied, the experimental magnetic flux density matches the simulated results qualitatively (Figure 9a). Here, negative *Bz*, relative to attractive magnetic force, becomes more negative for increasing size of central coil. Additional attraction is achieved with the inclusion of inter-windings. Vertical magnetic flux density is found independent of electrical connection configuration, as no significant differences are seen comparing SCR and MCR or SCRL and MCRL (Figure 9b). However, when comparing the S-configuration with the M-configuration, the resistance is seen higher for the M-configuration in both cases (CR and CRL), as these have longer electrical paths.

Additionally, MEMT with larger central coils (L-large) present larger amplitudes of *Bz*, as well as higher *Bz_,maximum_* and lower *Bz_,minimum_* (see SCR and SCRL or MCR and MCRL) with only 1 Ω increase in system resistance. The inclusion of inter-windings (CRIN and CRIF) clearly promoted a steep increase in *Bz* with no significant increase in resistance. 

The integration of opposite magnetic behaviors in the same MEMT and instantaneous reversing of current (the transient response of the power supply is of 0.01 s) leading to rapid reverse of magnetic flux density stands favorable for particle trapping and cell attraction/repulsion. CRIN MEMT promoted the largest amplitudes in *Bz* while at constant gradient and was therefore chosen to proceed to further experiments.

**Figure 10:**
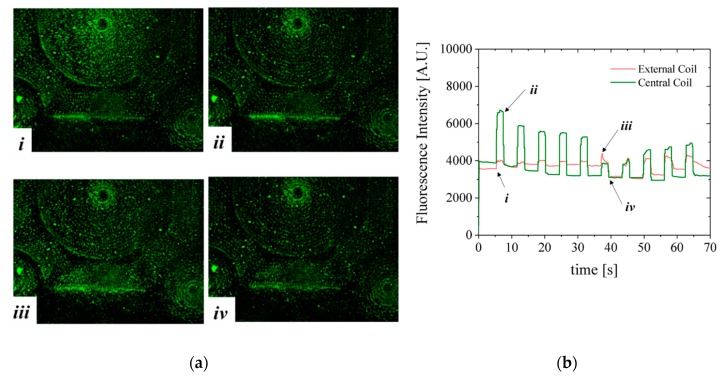
(**a**) Visualization of MB trapping and repulsion while actuating the CRIN MEMT design with 5 pulses of 2 s duration and 4 s interval with current +1 A followed by 5 pulses with current −1 A. (i) Start of +1 A pulse for 2 s duration. Synchronous attraction to the central coil and repulsion from the external coils is occurring; (ii) pulse stop with deactivation of current to 0 A. The fluorescence intensity of the central coil decreases abruptly; (iii) inversion of current to −1 A. The electromagnetic force generated is directing MB away from the central coil and attracting MB to the external coils; (iv) deactivation at 0 A; (**b**) MB fluorescence signal on top of one external coil and of the central coil deriving from MB actuation. MB are in direct contact with the MEMT. (Video S2: Multimedia view).

The manipulation of Estapor ^©^ superparamagnetic fluorescent beads by the CRIN MEMT design is depicted in Figure 10. The solution of beads is pumped through the channel. Lateral and top walls were added to the chip to obtain a microfluidic channel roughly 200 µm high, 2.5 mm wide and 10 mm long. In this configuration the MB are in direct contact with the MEMT. The MEMT is activated by running a sequence of 10 pulses of 2 s duration with current 1 A and 4 s interval. The current is inverted after the first 5 pulses. During the time of actuation, the fluorescence signal intensifies, to dim right after actuation stops.

The fluorescent nature of Estapor^®^ MB enabled direct observation of resulting magnetic actuation via fluorescence microscopy. The beads, once concentrated to the same area after MEMT actuation, are seen to agglomerate into larger diameter particulates and move faster in subsequent manipulations (Figure 10a(i,iii)). When the MEMT actuation is turned off, these particulates quickly disperse (Figure 10a(ii,iv)). Figure 10b shows MB fluorescence intensity over time of actuation for experiment depicted in Figure 10a. By the changes in fluorescence signal, the moments of attraction and repulsion and change in actuation signal for each position in the chip is clear. In this configuration, the beads tend to adhere to the spaces matching the shape of the MEMT tracks and agglomerate on top of those. The resulting adherence of particles to the coils after each pulse is perceived by the slight increase in the baseline of the fluorescence intensity for the external coil in the first 5 pulses and for the central coil in the last 5 pulses. The electromagnetic force directing MB away from the surface is not enough to overcome surface adhesion forces between the coil and the MB. This feature is particularly attractive to enhance surface binding in heterogeneous immunoassays in which one requires separation of bound and free labels after the biding reaction of a target substance (e.g., antigen) with the antibody [22]. 

**Figure 11:**
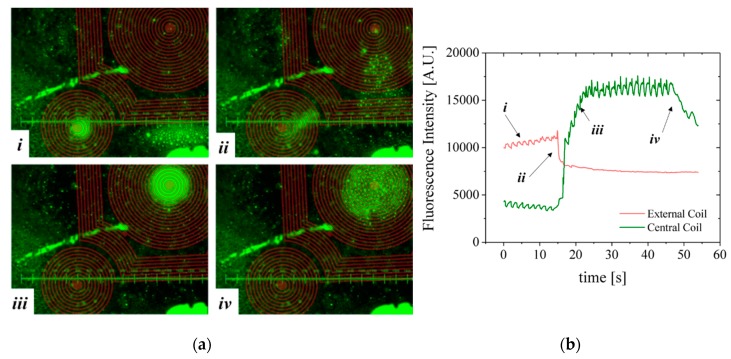
(**a**) Experimental results from CRIN MEMT 1 s activation/0.5 s deactivation pulses and resulting MB concentration. The current is inverted after 9 pulses. (i) 9 pulses activation period of −1 A; (ii) inversion of the current to +1 A; (iii) increasing attraction of MB to the central coil; (iv) sequence stop. (**b**) MB fluorescence signal over time of actuation. Separation between the MEMT and the channel bottom: 100 μm. (Video S3: multimedia view).

A second configuration, more favorable to homogeneous immunoassays [23], was mounted comprising the microfluidic channel with 100 µm-thick bottom wall placed on top of the MEMT forcing the separation between the MB and the MEMT (Figure 11). As such, images are focused from 0.2 to 0.5 mm away from the MEMT surface. Because the coils are not focused on the image, a brown CAD overlay is aligned with the MEMT tracks for easier analysis of the actuation. In this experiment, the activation is done by running a sequence of 29 pulses of 1 s duration with current 1 A and 0.5 s interval and inverting the current after the first 9 pulses.

Although the MB are subjected to lower magnetic flux densities at 100 µm from the MEMT, their distribution is uniform on top of the MEMT, preventing local agglomeration and adhesion to the chip walls (Figure 11a), also perceived from the MB fluorescence signal (Figure 11b). The brighter green color seen on top of the tracks, which could indicate bead—coil surface adhesion, is merely an artifice caused by the inclusion of the brown CAD overlay in this case. 

During the first 9 pulses of −1 A, there is synchronous repulsion from the central coil and attraction to the external coils (*i*). When the current is inverted to +1 A, the fluorescence intensity inverts (*ii*) due to the increasing attraction of MB to the central coil and their repulsion from the external coils (*iii*). After the actuation sequence stops, the MB disperse (*iv*) due to Brownian and viscous forces. The line-type arrangement of MB remaining on the left side of the image is a result of bead-top wall surface adhesion. Effective concentration or dispersion of MB is better noticed by the variation of the base plateau after actuation pulses. 

Not only did systems with inter-windings between the central and lateral coils (CRIN and CRIF) present the highest difference between *B_attractive_* and *B_repulsive_* but the magnetic force generated by the inter-windings promoted the movement of the MB from coil to coil, more specifically, by being able to pull MB from the inter-winding area. This was also foreseen in simulated results (Figure 7e) and magnetic scans of the MEMT surface (Figure 9a) by the steep slope obtained in the inter-winding region of the CRIN and CRIF MEMT. Nonetheless, the magnetic force fields need to overcome the hydrodynamic forces and adhesion forces present in the actual functionalization of channel surfaces to become effective in the manipulation and concentration of MB.

## 4. Conclusions

The development of immunoassays is dependent on well-defined antibody capture and detection strategies. Microelectromagnets (MEMT) for magnetic trapping and manipulation of functionalized superparamagnetic beads promotes the development of biomedical and biological microfluidic systems as it increases the capabilities of continuous immunoassays by improving immobilization, concentration and biding of bioanalytes in specific regions.

Six different MEMT designs were simulated, fabricated, and tested to evaluate the influence of MEMT configuration and coil positioning and dimensions on the concentration and dispersion performance of MB in a bioassay. The designs proposed in this experimental campaign all showed consistency in synchronous attraction and repulsion of MB. Inter-windings between the central coil and external coils (CRIN and CRIF) increased magnetic flux density and promoted coil-to-coil movement consequently increasing the capture/dispersion yield. CRIN and CRIF more than doubled the difference between *B_attractive_* and *B_repulsive_* when compared to the MEMT with no inter-windings (SCRL and MCRL). CRIN MEMT showed a 210% increase on *B_attractive_* when compared to MCRL, as an example. Overall, the conception and optimization implemented in this work allowed successful trapping both for homogeneous and heterogeneous immunoassays potentiating this technology towards novel TDM strategies.

## Supplementary Materials

The following are available online at https://www.mdpi.com/2072-666X/10/9/607/s1, Video S1: multimedia view Figure 1; Video S2: multimedia view Figure 10; Video S3: multimedia view Figure 11

## Appendix A

### Appendix A.1. Magnetic Force on a Magnetic Dipole due to a Static Magnetic Field

In a magnetic field $\vec{B}$, given a superparamagnetic punctual dipole of moment $m=f\left(\left|\vec{B}\right|\right)\hat{B}$, where f is defined by $f\left(x\right)=V\cdot {\left|\vec{M}\right|}_{\left|\vec{B}\right|=x}\;$, its magnetic potential is

(A1) $$\vec{U}=-\vec{m}.\vec{B}=-f\left(\left|\vec{B}\right|\right)\left|\vec{B}\right|$$

and the magnetic force given by

(A2) $${\vec{F}}_{m}=-\nabla U\;=\nabla \left(\left(id\cdot f\right)\circ \left|\vec{B}\right|\right)={\left(id\circ f\right)}^{\prime }\left(\left|\vec{B}\right|\right)\cdot \nabla \left|\vec{B}\right|=\;\left(f+id\cdot f^{\prime }\right)\left(\left|\vec{B}\right|\right)\cdot \nabla \left|\vec{B}\right|$$

where id is a function defined by id(x):=x.

Considering

(A3) $$\nabla \left|\vec{A}\right|=\frac{\nabla {\left(\left|\vec{A}\right|\right)}^{2}}{2\left|\vec{A}\right|}$$

and

(A4) $$\nabla \left(\vec{A}.\vec{A}\right)=\underset{i,j}{\sum }\hat{i}\partial_{i}\left(A_{j}A_{j}\right)=\underset{i,j}{\sum }2\hat{i}A_{j}\partial_{i}A_{j}=2\left[\nabla A_{j}\right]\vec{A}=2\;{\left(\nabla \vec{A}\right)}^{T}\vec{A}=2\left(\nabla \vec{A}\right)\cdot \vec{A}$$

where $\nabla \vec{A}:=\left[\partial_{j}A_{i}\right]$ and $\partial_{i}:=\frac{\partial }{\partial r_{i}}$ with $\vec{r}=\left(x,y,z\right)$, we obtain

(A5) $$\nabla \left|\vec{B}\right|=\frac{\nabla {\left(\left|\vec{B}\right|\right)}^{2}}{2\left|\vec{B}\right|}=\frac{\nabla {\left(\vec{B}.\vec{B}\right)}^{2}}{2\left|\vec{B}\right|}=\frac{\left(\nabla \vec{B}\right)\cdot \vec{B}}{\left|\vec{B}\right|}$$

Combining the above, the magnetic force is given as

(A6) $$\begin{array}{l} {\vec{F}}_{m}=\;\left(f+id\cdot f^{\prime }\right)\left(\left|\vec{B}\right|\right)\cdot \nabla \left|\vec{B}\right|=\;\left(f\left(\left|\vec{B}\right|\right)\right)+\left|\vec{B}\right|\cdot f^{\prime \prime }\left(\left|\vec{B}\right|\right).\frac{\left(\nabla \vec{B}\right)\cdot \vec{B}}{\left|\vec{B}\right|}\\ \;=\nabla \vec{B}\cdot \vec{B}\cdot \left(f^{\prime }\left(\left|\vec{B}\right|\right)+\;\frac{f\left(\left|\vec{B}\right|\right)}{\left|\vec{B}\right|}\right)=V\cdot \nabla \vec{B}\cdot \vec{B}\cdot \left(M^{\prime }\left(\left|\vec{B}\right|\right)+\frac{M\left(\left|\vec{B}\right|\right)}{\left|\vec{B}\right|}\right) \end{array}$$

M is the uniform magnetization magnitude inside the particle.

### Appendix A.2. Field Produced by Cuboid of Uniform Current Density

The magnetic field $\vec{B}$ at a point ${\vec{r}}_{o}$ is produced by a cuboid, i.e., a parallelepiped of straight angles, of length a, width b and height *c*, through which passes a uniform current density $\vec{J}$. Let $\vec{s}:=\left(a,b,c\right)$ and corners at $\vec{s}/2$ and $-\vec{s}/2$. From Bio-Savart’s formula, we obtain:

(A7) $$\vec{B}=\;\frac{\mu_{0}}{4\pi }\vec{J}\times \int_{-a/2}^{a/2}\int_{-b/2}^{b/2}\int_{-c/2}^{c/2}\left(\frac{{\vec{r}}^{\prime }}{{\left|{\vec{r}}^{\prime }\right|}^{3}}dx^{\prime }dy^{\prime }dz^{\prime }\right)$$

where ${\vec{r}}^{\prime }=\;{\vec{r}}_{o}-\vec{r}$ and $\vec{r}=\left(x,y,z\right)$.

Let $\vec{U}$ be the right factor of the cross-product. Using symbolic calculation to obtain the indefinite integral $\vec{u}\left(x,y,z\right)={\int \int \int }^{\;}\frac{\vec{r}}{{\left|\vec{r}\right|}^{3}}dV$ in closed form we can obtain $\vec{U}=\;\vartheta \vec{u}$, where

(A8) $$\vartheta f:=-\overset{2}{\underset{i=1}{\sum }}\overset{2}{\underset{j=1}{\sum }}\overset{2}{\underset{k=1}{\sum }}{\left(-1\right)}^{i+j+k}.(f{|}_{\begin{array}{c} x\leftarrow x_{o}-{\left(-1\right)}^{i}a/2,\\ \;y\leftarrow y_{o}-{\left(-1\right)}^{j}b/2,\\ \;z\leftarrow z_{o}-{\left(-1\right)}^{k}c/2) \end{array}}$$

Here a←b indicates symbolic replacement of a by b, $\left(x_{o},y_{o},z_{o}\right):={\vec{r}}_{o}$, and the leading minus is required by the change of variable from $\vec{r}$ to $\vec{r}=\;{\vec{r}}_{o}-{\vec{r}}^{\prime }$.

Let $P_{S}$ be the operator that permutes the symbols {x,y,z} according to a permutation S of 3 elements. Let $S_{i}$ be the cyclic permutation of x,y,z that map x to $r_{i}$. By symmetry, we obtain $\vartheta {\vec{u}}_{i}=\vartheta P_{S_{i}}{\vec{u}}_{1}$ (mathematical conventions use indexes 1,2,3 to indicate x,y,z).

Any term in $\vec{u}$ that depends only on two of the variables x,y,z is nulled by the summation performed by ϑ. After removing those terms from the symbolic calculation results, $\vec{u}=Ph$ can be used. Here h is a scalar expression in x,y,z, and P is an operator defined by $Pf=\left(P_{S_{1}}f,P_{S_{2}}f,P_{S_{3}}f\right)$, i.e., taking the expression and construct a 3-vector by cyclically permuting x,y,z.

Calculating the gradient yields

(A9) $$\nabla \vec{B}=\left[\partial_{j}B_{i}\right]=\left[\partial_{j}\vec{B}\right]=\left[\frac{\mu_{0}}{4\pi }\vec{J}\times \partial_{j}\vec{U}\right]:=\frac{\mu_{0}}{4\pi }\vec{J}\times \left[\partial_{j}\vec{U}\right]$$

by defining the cross-product of a vector by a matrix as $\vec{a}\times M:=\left[\vec{a}\times col_{j}M\right]$. Hence

(A10) $$\left[\partial_{j}\vec{U}\right]=\left[\partial_{j}\vartheta \vec{u}\right]=\vartheta \left[\partial_{j}\vec{u}\right]=\vartheta \left[\partial_{j}Ph\right]=\vartheta \left[\partial_{j}P_{S_{i}}h\right]=\vartheta \left[P_{S_{i}}\partial_{s_{i}^{-1}\left(j\right)}h\right]=\vartheta \left[P_{S_{i}}{\left(\nabla h\right)}_{s_{i}^{-1}\left(j\right)}\right]$$

Calculating ∇h, and eliminating terms with only two variables as for $\vec{u}$, we can obtain $\vec{g}$.

Both h and $\vec{g}$ can be written in terms of $n:=\left|\vec{r}\right|$, $w:=P\arctan\left(\frac{yz}{xn}\right)$ and $\vec{l}:=P\ln\left(x+n\right)$. Then

(A11) $$\vec{g}=\left(w_{x},-l_{z},-l_{y}\right),\;h=\vec{r}\cdot \vec{g}$$

and

(A12) $$\left[P_{S_{i}}{\vec{g}}_{s_{i}^{-1}\left(j\right)}\right]=\left[\begin{array}{ccc} w_{x} & -l_{z} & -l_{y}\\ -l_{z} & w_{y} & -l_{x}\\ -l_{y} & -l_{x} & w_{z} \end{array}\right]=:G\left(\vec{w},\vec{l}\;\right)$$

G is linear in $\vec{w}$ and $\vec{l}$. Therefore

(A13) $$\left[\partial_{j}\vec{U}\right]=\vartheta P\left({\vec{g}}^{T}\right)=\vartheta G\left(\vec{w},\vec{l}\;\right)=G\left(\vartheta \vec{w},\vartheta \vec{l}\;\right)$$

Finally,

(A14) $$\vec{B}=\frac{\mu_{0}}{4\pi }\vec{J}\times \vartheta P\left(\vec{r}\cdot \vec{g}\right)\;,\;\;\;\;\;\nabla \vec{B}=\frac{\mu_{0}}{4\pi }\vec{J}\times G\left(\vartheta \vec{w},\vartheta \vec{l}\;\right)$$

Identifying these patterns enabled building efficient code for the calculation of both the magnetic field and its gradient.
